# A Novel Approach Using Reduced Graphene Oxide for the Detection of ALP and RUNX2 Osteogenic Biomarkers

**DOI:** 10.3390/cimb46050272

**Published:** 2024-05-08

**Authors:** Elena Alina Chiticaru, Mariana Ioniță

**Affiliations:** 1 Faculty of Medical Engineering, National University of Science and Technology Politehnica Bucharest, Gh. Polizu 1-7, 011061 Bucharest, Romania; elena.chiticaru@upb.ro; 2 Advanced Polymer Materials Group, National University of Science and Technology Politehnica Bucharest, Gh. Polizu 1-7, 011061 Bucharest, Romania

**Keywords:** reduced graphene oxide, osteogenic biomarkers, ALP, RUNX2, electrochemistry, electrode modification

## Abstract

In this work, we propose a new technique involving the modification of commercial screen-printed carbon electrodes with electrochemically reduced graphene oxide to serve as the starting point of a future electrochemical biosensor for the detection of two osteogenic biomarkers: alkaline phosphatase (ALP) and Runt-related transcription factor 2 (RUNX2). The electrodes were characterized after each modification by cyclic voltammetry and electrochemical impedance spectroscopy, showing the appropriate electrochemical characteristics for each modification type. The results obtained from scanning electron microscopy, Raman spectroscopy, X-ray photoelectron spectroscopy, and contact angle measurements are well correlated with each other, demonstrating the successful modification of the electrodes with graphene oxide and its subsequent reduction. The bioreceptors were immobilized on the electrodes by physical adsorption, which was confirmed by electrochemical methods, structural characterization, and contact angle measurements. Finally, the functionalized electrodes were incubated with the specific target analytes and the detection relied on monitoring the electrochemical changes occurring after the hybridization process. Our results indicated that the pilot platform has the ability to detect the two biomarkers up to 1 nM, with increased sensitivity observed for RUNX2, suggesting that after further optimizations, it has a high potential to be employed as a future biosensor.

## 1. Introduction

The field of biosensing technology has witnessed remarkable advancements in recent years, driven by the ever-growing demand for rapid, sensitive, and selective detection of biomolecules. Among the diverse applications of biosensors, the monitoring of biomarkers associated with bone health and osteogenic processes has gained significant attention due to its vital role in the early diagnosis and management of skeletal disorders and bone-related diseases [1,2]. Alkaline phosphatase (ALP) and Runt-related transcription factor 2 (RUNX2) are pivotal osteogenic biomarkers, each serving as a critical indicator of cellular osteogenic differentiation and bone formation [3,4].

ALP, an enzyme found in various tissues throughout the body, is particularly abundant in bone tissue. Its significance lies in its direct involvement in the mineralization of bone matrix [5], a process vital for bone strength and density [6]. ALP is not only an indicator of osteoblast activity but also serves as an essential catalyst for the conversion of inorganic pyrophosphate to inorganic phosphate, a critical step in hydroxyapatite crystal formation within the bone matrix [7]. Thus, the quantification of ALP levels in biological samples provides valuable insights into osteoblast functionality and bone mineralization, an important factor in bone regeneration applications [8,9], and aids in the diagnosis and monitoring of skeletal disorders such as osteoporosis, Paget’s disease, and osteomalacia.

In conjunction with ALP, the transcription factor RUNX2 plays a central role in osteogenesis [10,11]. RUNX2, also known as core-binding factor alpha 1 (Cbfa1), orchestrates the differentiation of mesenchymal stem cells into osteoblasts, the primary bone-forming cells [12]. Through its regulatory influence on osteogenic genes, RUNX2 directs the synthesis of bone matrix proteins, including collagen type I and osteocalcin [13,14], facilitating bone tissue development and remodeling. Its pivotal role in osteoblast differentiation and bone formation makes RUNX2 a cornerstone biomarker in the study of bone metabolism, development, and regeneration.

The reference ranges for ALP levels in the blood can vary between laboratories, but in general, normal levels are often considered to be between 20 and 140 international units per liter (IU/L) for adults [15]. However, it is noted that ranges can differ based on factors such as age, gender, and specific assay methods used by different laboratories [16]. Quantifying specific normal or pathological levels in the blood is not as common for RUNX2, because it is predominantly a transcription factor involved in gene regulation. It is often assessed at the messenger RNA (mRNA) level (gene expression), rather than as a circulating protein. Therefore, numerical reference ranges may not be as applicable in the context of RUNX2 compared to enzyme levels like ALP.

The intricate interplay between physiological and pathological levels of ALP and RUNX2 holds profound implications for cellular and molecular processes. In a physiological context, ALP, a pivotal enzyme, is prominently associated with bone formation, wherein elevated levels denote active osteoblastic activity and matrix mineralization [5,7]. Concurrently, RUNX2, a master regulator of osteogenesis, orchestrates the differentiation of mesenchymal stem cells into osteoblasts, contributing to skeletal development and homeostasis [17,18]. However, deviations from these physiological levels may herald pathological conditions. Elevated ALP levels may signify disorders such as liver disease, bone disorders, or malignancies, reflecting aberrations in bone metabolism and tissue calcification [16]. Similarly, dysregulation of RUNX2 expression has been implicated in skeletal disorders and neoplastic processes, where its overexpression or underexpression may disrupt bone homeostasis [19]. A nuanced understanding of the dynamic balance between physiological and pathological levels of ALP and RUNX2 biomolecules is crucial for unraveling their intricate roles in health and disease, providing insights into potential diagnostic and therapeutic avenues for conditions ranging from metabolic bone diseases to cancer.

Traditionally, the quantification of ALP and RUNX2 has relied on laborious and often expensive techniques, including enzyme-linked immunosorbent assays (ELISAs) [20,21], Western blotting [22,23], and polymerase chain reaction (PCR) [24,25]. These methods, while valuable for research, are impractical for rapid, point-of-care diagnostics or real-time monitoring of osteogenic processes. To address the growing demand for efficient, high-throughput, and sensitive detection of ALP and RUNX2, scientists have turned their attention toward innovative biosensing technologies, among which graphene-based biosensors stand out as a pioneering solution. In particular, reduced graphene oxide (RGO), a derivative of graphene, has emerged as a star player in this field, owing to its remarkable properties. RGO exhibits excellent electrical conductivity [26], a large surface area [27], and facile functionalization capabilities [28], making it an ideal candidate for biosensor development. These properties enable the precise immobilization of biorecognition elements such as antibodies [29,30], aptamers [31,32], or enzymes [33] onto the RGO surface, facilitating selective and highly sensitive biomarker detection.

The detection of osteogenic biomarkers is of great importance in various domains of health care and research. Not only it can enable early detection of bone-related diseases such as osteoporosis, osteoarthritis, and bone metastases, which is essential, as it allows timely intervention and treatment that can stop or slow disease progression, but it can also lead to personalized treatment plans for patients based on the individual’s biomarker profile. Moreover, osteogenic biomarker detection has great implications in stem cell treatments for bone defects, as it can predict stem cell differentiation into osteoblasts, and therefore it can indicate if the treatment will be successful, avoiding costly procedures that are unlikely to succeed and improving patient care.

So far, there have been a few studies focusing on the detection of either ALP or RUNX2 with the help of graphene-based biosensors. Mahato et al. [34] developed an impedimetric biosensor using gold-nano-dendroids and graphene oxide (GO) detecting ALP in human serum samples, Liu et al. [35] describe a fluorescent assay based on GO and λ exonuclease for ALP activity, while to the best of our knowledge, RUNX2 detection is still performed using classical techniques like quantitative PCR (qPCR) [36], Western blot [37], and immunohistochemistry [38], while no progress has been made regarding the development of a novel electrochemical biosensor to detect this biomarker. Ma’arif et al. [22] compared two techniques used for RUNX2 detection in bone tissue, i.e., Western blot and immunohistochemistry, investigating 70 articles, and concluded that the Western blot method is superior in terms of sensitivity, selectivity, cost, and detection time. However, neither of these techniques gives a fast response, the Western blot method having a process time over 25 h, while immunohistochemistry takes over 46 h to generate a result. Therefore, we believe there is a need for an improved biomarker detection method that can contribute to the development of point-of-care devices, designed to significantly decrease the costs and response time while preserving the high selectivity and sensitivity standards of the traditional methods.

In this context, our paper presents a pilot study and focuses on the design phase and the identification of key parameters required to fabricate in the end a biosensor for ALP and RUNX2 detection. Our prior work paved the way for the current study, where we have specifically tailored the application towards detecting these specific osteogenic biomarkers, and this new direction represents a distinctive contribution, as our earlier efforts primarily centered on generic DNA sequences [39,40,41]. Therefore, we show that modifying screen-printed carbon electrodes (SPCEs) with RGO can lead to the design of a novel detection platform for specific biomarkers, i.e., ALP and RUNX2, with utmost importance for bone regeneration processes. The fabricated platform has been characterized by scanning electron microscopy (SEM), Raman spectroscopy, X-ray photoelectron spectroscopy (XPS), contact angle measurements, and electrochemical techniques, such as cyclic voltammetry (CV) and electrochemical impedance spectroscopy (EIS). The detection of certain biomarkers, i.e., RUNX2 and ALP, signifies a strategic advancement, aiming to develop a biosensor for a specific application that is not only robust but also cost-effective. The nuanced modifications in the experimental design and target molecules align with our previous commitment to diversifying the applications of our biosensor technology.

## 2. Materials and Methods

### 2.1. Reagents and Materials

Graphene oxide dispersion in water at a concentration of 2 mg/mL (code: 763705), KCl (CAS no.: 7447-40-7, 99–100.5% purity), HCl (CAS no.: 7647-01-0, 36.5–38% purity), H_2_NaO_4_P (CAS no.: 13472-35-0, ≥99% purity), and HNa_2_O_4_P (CAS no.: 7558-79-4, 98–100.5% purity) were purchased from Sigma-Aldrich (St. Louis, MO, USA). Potassium ferricyanide (K_3_[Fe(CN)_6_], CAS no.: 13746-66-2, ≥99% purity) and potassium ferrocyanide (K_4_[Fe(CN)_6_]·3H_2_O, CAS no.: 14459-95-1, 99–102% purity) were procured from Merck Co. (Darmstadt, Germany). From Integrated DNA Technologies, Inc. (Coralville, IA, USA) were purchased the bioreceptors for ALP (5′-CGT CAC TCT CAT ACT CCA CAT C-3′) and RUNX2 (5′-GAC GGT TAT GGT CAA GGT GAA-3′), as well as the target molecules for both biomarkers (ALP: 5′-G ATG TGG AGT ATG AGA GTG ACG-3′, RUNX2: 5′-TTC ACC TTG ACC ATA ACC GTC-3′). Following each modification, the SPCEs were thoroughly rinsed with ultrapure water obtained from a water purification system (Adrona Crystal EX, Riga, Latvia), which has a resistivity of 18.2 MΩ·cm. SPCE-DRP 110 electrodes were purchased from Metrohm DropSens, Oviedo, Spain.

### 2.2. Procedures

#### 2.2.1. Electrochemical Measurements

The characterization of each modification of SPCEs was performed by CV and EIS at room temperature using a potentiostat/galvanostatAutolab PGSTAT 204 (Metrohm Autolab, Utrecht, The Netherlands), which was equipped with NOVA 2.1 software. For the electrochemical measurements, a 100 µL volume of a 0.1 M KCl electrolyte solution containing a 1 mM [Fe(CN)_6_]^3−/4−^ redox probe was added onto the SPCE. CV curves were acquired by scanning the potential within the range of −0.2 V to +0.6 V at a sweep rate of 0.05 V/s unless otherwise specified. Impedance spectra were recorded across a frequency span of 0.01–10^5^ Hz, employing a 10 mV AC amplitude at the formal potential of the [Fe(CN)_6_]^3−/4−^ redox system relative to the Ag pseudo-reference electrode. The data from CV and EIS was plotted using the software OriginPro 8.1 (OriginLab Corp., Northampton, MA, USA).

#### 2.2.2. Morphological and Structural Characterization

To investigate the morphology of the electrodes following each modification, an FEI high-resolution focused ion beam scanning electron microscopy (FIB-SEM) system, specifically the Versa 3D DualBeam model from FEI Company in Hillsboro, OR, USA, was employed. This system was operated to examine the surface morphology of the plane view (0° tilt) samples. Surface morphology analysis was conducted by detecting secondary electron (SE) signals in high-vacuum operation mode with a vacuum level of 6.1 × 10^−4^ Pa. A working distance of 10 mm, an accelerating voltage of 10 kV, and a spot size of 4.5 were chosen for this analysis. To ensure stable imaging, the SmartSCAN scanning strategy and DCFI drift suppression features of the Versa 3D DualBeam tool were employed.

Furthermore, Raman spectroscopy was employed to confirm the reduction of graphene oxide by investigating the structural changes on the electrode surface. Raman spectra were acquired using a Renishaw in Via Raman confocal spectrometer, from Brno-Černovic, Czech Republic. The laser excitation wavelength used for these measurements was 473 nm, and a 100× objective was employed for data collection.

#### 2.2.3. Wettability Investigations

The wettability assessment of the samples was carried out by the Drop Shape Analyzer DSA100 (Krüss Scientific GmbH, Hamburg, Germany) using the sessile drop method and Young–Laplace equation. After deposition of a water drop on the sample surface, the shape of the droplet with a volume of 2 μL was recorded with a CF03 digital camera for 10 s at room temperature. The values of the static water contact angle were calculated using Advance software 1.7.2.1. and are presented as the average values of three measurements along with the standard deviation.

#### 2.2.4. Preparation and Detection Testing of the Modified SPCEs

The electrodes, denoted RGO/SPCEs after being modified with electrochemically reduced graphene oxide, were prepared following a well-established procedure outlined previously by us [39]. Before any modification, the SPCEs underwent a pretreatment process involving five voltammetric cycles carried out in a 0.1 M HCl solution. These cycles spanned from +0.5 to −1.5 V, and the scan rate employed was 0.05 V/s. Subsequently, another set of five cycles took place in a 0.1 M phosphate buffer solution (PBS) at pH 7, ranging from 0 to +2 V, also at a scan rate of 0.05 V/s.

Following the activation step, 3 µL of PBS was meticulously deposited onto the working electrode (WE) surface of the SPCEs. Subsequently, the electrodes underwent a thorough wash with ultrapure water and were dried at 60 °C. Altering the surface properties of the carbon substrate effectively ensures a consistent and reproducible GO modification, representing an extremely important step, as detailed in our previous study [39].

Once the SPCEs had cooled to room temperature, a volume of 3 µL of GO dispersion, at a concentration of 0.3 mg/mL, was evenly cast onto their surface. The modified electrodes were then subjected to a drying process at 60 °C for a duration of 2 h, followed by an overnight rest period at room temperature. To complete the process, the GO/SPCEs underwent electrochemical reduction through ten CV cycles, which involved applying a potential that ranged from 0 to −1.5 V at a scan rate of 0.1 V/s in the presence of a 0.5 M KCl solution.

Both ALP and RUNX2 bioreceptors were immobilized on the RGO/SPCE surface (different electrodes) by incubating them with 10 µL single-stranded DNA (ssDNA) as probe using a concentration of 1 µM at room temperature overnight. To test the analyte detection, the fabricated biosensors were then incubated with 1, 10, 50, and 100 nM ssDNA target corresponding to ALP and RUNX2, respectively, at 46 °C for 2 h. To ensure the robustness and reproducibility of the procedure, the experiments were conducted in triplicate.

## 3. Results and Discussion

**Figure 1:**
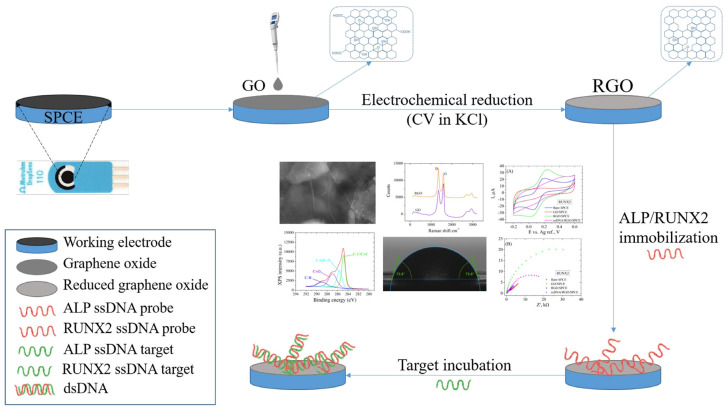
Schematic representation of the electrochemical detection platform fabrication process.

In the current paper, we present the fabrication of an electrochemical platform designed to detect specific osteogenic biomarkers, such as ALP and RUNX2. We started from commercial electrodes, i.e., SPCEs, that we modified with GO that was electrochemically reduced using the CV technique. The bioreceptors (ALP probe and RUNX2 probe) were immobilized on RGO-modified electrodes by physical adsorption, then the electrodes were incubated with the target biomarkers, which were detected electrochemically. Electrode modifications were characterized by SEM, Raman spectroscopy, XPS, contact angle measurements, CV, and EIS. A schematic representation of the fabrication process step by step is presented in Figure 1.

### 3.1. Morphological Characterization

**Figure 2:**
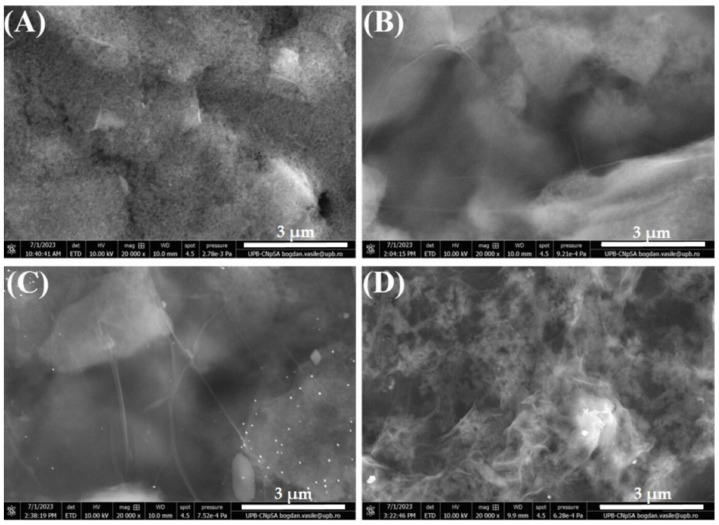
SEM images of bare (**A**) SPCE, (**B**) GO/SPCE, (**C**) RGO/SCPE, and (**D**) ssDNA/RGO/SPCE. Images recorded at 20 kX magnification (3 µm scale).

SEM was performed to assess the surface morphology of the modified electrodes. First, a bare SPCE was investigated (Figure 2A) to serve as a baseline for comparison with subsequent modifications. The image shows a clean, unmodified surface, typical for this substrate as observed in our previous studies [39,40]. Figure 2B presents the electrode modified with GO, showing thin (probably just few stratum of GO sheets), wrinkled, transparent layers covering the carbon electrode, a morphology that does not suffer drastic changes after GO electrochemical reduction, as observed in Figure 2C. However, the subsequent immobilization of ssDNA (ALP bioreceptor) on the RGO substrate determines a profound alteration in the surface morphology (Figure 2D), indicating that the incubation procedure was successful in immobilizing the bioreceptor on the modified electrode.

### 3.2. Structural Characterization

**Figure 3:**
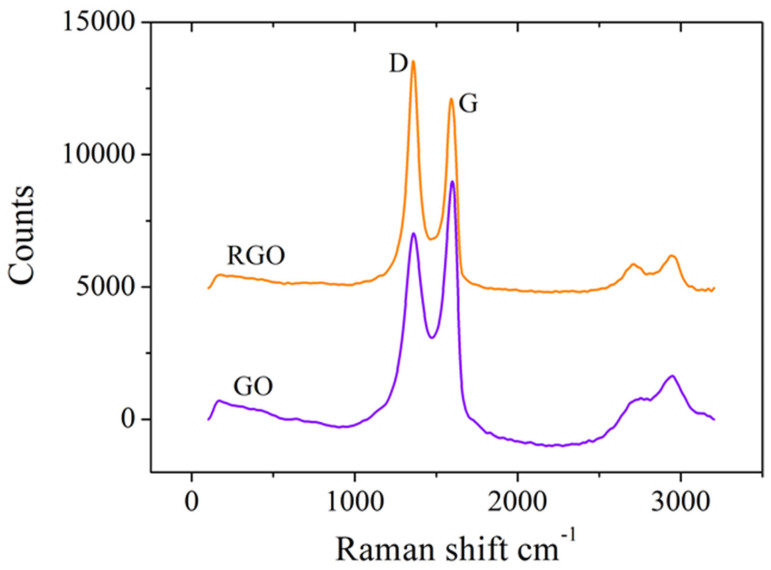
Raman spectra of SPCEs modified with GO and RGO.

Raman spectroscopy analysis was used to confirm the successful electrochemical reduction of GO by assessing the structural changes within the graphene material. The results (Figure 3) show that the D and G vibrational bands corresponding to GO are situated at 1359 cm^−1^ and 1597 cm^−1^, respectively, and they do not shift considerably for RGO. However, the intensity ratio of the D and G bands (I_D_/I_G_) increased from 0.77, measured for GO, to 1.22 in the case of RGO, clearly indicating an elevated level of defects in the structural framework of RGO caused by the reduction in graphitic domains when functional groups were removed from the GO surface. These results are not only confirmed by our previous studies [39,40] but also by other research groups like Das et al. [42] and Bharath et al. [43].

**Figure 4:**
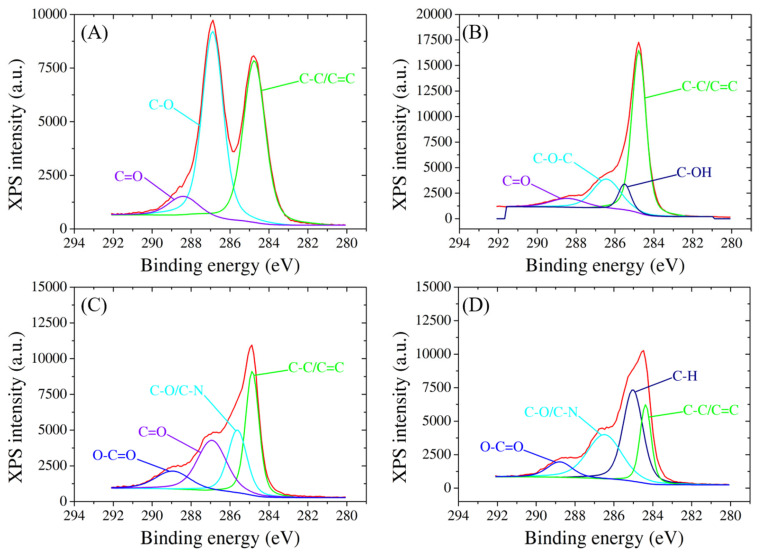
High-resolution C1 XPS spectra of SPCE modified with (**A**) GO, (**B**) RGO, (**C**) probe, and (**D**) target molecules.

XPS analysis also offers comprehensive insights into the surface chemistry and compositional changes of SPCEs as it undergoes a series of modifications with GO, RGO, probe ssDNA, and target biomarker. The high-resolution XPS spectra reveal distinctive peaks corresponding to the binding energies of carbon, oxygen, and nitrogen species, confirming the presence and successful integration of each component onto the electrode surface (Figure 4). For the C1 species, GO modification introduces peaks at 284.7 eV, 286.9 eV, and 288.4 eV, indicative of C-C/C=C, C-O, and C=O bonds associated with the graphene oxide structure (Figure 4A). Subsequent RGO modification results in peaks at 284.7 eV (C-C/C=C), 285.5 eV (C-OH), 286.4 eV (C-O-C), and 288.4 eV (C=O) [44], demonstrating the reduction in oxygen functionality and the emergence of sp2 carbon structures (Figure 4B). Notably, the immobilization of ssDNA on the RGO-modified electrode introduces additional changes in the C1 spectrum, recording peaks at 284.8 eV, 285.6 eV, 286.9 eV, and 288.9 eV, corresponding to C-C/C=C, C-O/C-N, C=O, and O-C=O bonds, respectively [45] (Figure 4C). The hybridization process with the target further modifies the peaks to 284.3 eV (C-C/C=C), 285 eV (C-H), 286.4 eV (C-O/C-N), and 288.8 eV (O-C=O), indicating the stable integration of the target molecule onto the modified electrode (Figure 4D).

**Figure 5:**
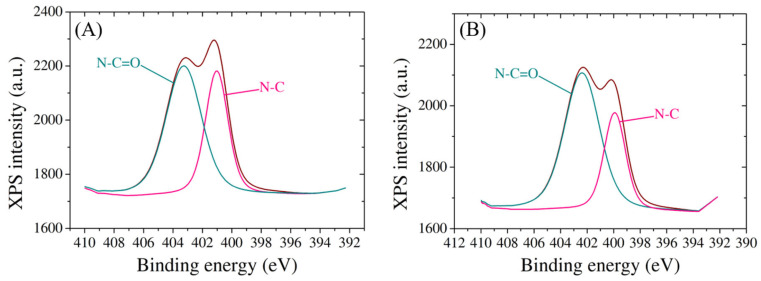
High-resolution N1 XPS spectra of SPCE modified with (**A**) probe molecule and (**B**) target molecule.

Moreover, the nitrogen species exhibit characteristic peaks, providing further evidence of the successful incorporation of biomolecules onto the electrode surface (Figure 5). The presence of both probe and target molecules is discernible through unique nitrogen peaks, offering a quantitative understanding of the density of DNA binding. Two spectra for N1 species show peaks at 401 eV and 403.2 eV after probe immobilization, attributed to N-C and N-C=O bonds, respectively [46] (Figure 5A), and the same bonds appear following the hybridization with target at 400 eV and 402.3 eV (Figure 5B), suggesting that the target molecule remained attached on the electrode surface. The observed shifts in binding energies and relative peak intensities underscore the intricate interactions between the electrode and the modified layers, further contributing to the fundamental understanding of the tailored surface properties.

### 3.3. Wettability Investigations

The wettability properties of electrodes assume a pivotal role in the design and functionality of electrochemical biosensors for biomarker detection, where specific gene sequence recognition is paramount. The wettability of the electrode surface profoundly influences the success of molecular interactions essential for the biosensor’s performance.

**Figure 6:**
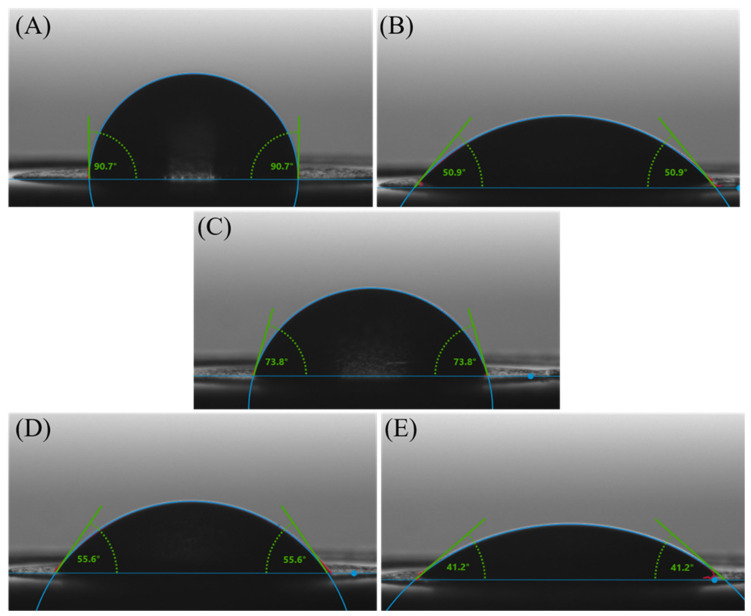
Contact angle variation on (**A**) unmodified SPCE, (**B**) GO/SPCE, (**C**) RGO/SPCE, (**D**) ssDNA/RGO/SPCE, and (**E**) dsDNA/RGO/SPCE.

The wettability characteristics of the electrodes used in this study are presented in Figure 6. Wettability is often characterized by the contact angle formed by a liquid droplet on the surface of a material. In Figure 6A, the 90.7° ± 3.28° water contact angles on the carbon electrode indicate a hydrophobic surface, with water droplets forming relatively spherical shapes, displaying limited wetting and spreading behavior. This hydrophobic behavior is common for many carbon-based materials due to their nonpolar nature and lack of functional groups [47] that promote strong interactions with water molecules.

For the electrodes modified with GO (Figure 6B), the water contact angles are 50.9° ± 2.63°, indicating moderate wettability. This property is a result of the unique surface characteristics of GO, which is derived from graphene through the introduction of oxygen-containing functional groups. These functional groups can render most areas of the GO surface hydrophilic while keeping others hydrophobic [48].

For the RGO-modified electrodes (Figure 6C), the water contact angles of 73.8° ± 0.52° indicate a moderately hydrophobic surface due to fewer oxygenated groups than GO, in agreement with Raman investigations that indicated changes in the chemical structure after the electrochemical reduction process. Eliminating oxygen-containing functional groups from the GO surface to obtain RGO resulted in a less polar and more hydrophobic material than GO. With the decreased polarity and oxygen content, RGO exhibits a surface that is less prone to wetting and adsorption of water molecules.

Upon binding the ssDNA bioreceptor to the RGO/SPCE (Figure 6D), the water contact angle decreased to 55.9° ± 1.17°, indicating that the probe adsorbed onto the RGO-modified electrodes, causing a slight increase in the surface hydrophilicity compared to bare RGO. These changes in wettability upon probe immobilization are in good correlation with the results obtained from structural characterization of the platform.

By immobilizing the complementary structure to the ssDNA probe, the water contact angle of 41.2° ± 1.2° on electrodes modified with RGO and double-stranded DNA (dsDNA) (Figure 6E) indicates a notably hydrophilic surface. The contact angle of 41.2° indicates a significant increase in the surface hydrophilicity after incubation of the functionalized SPCE with the target molecule compared to bare RGO. At the same time, an increase in the surface hydrophilicity is also observed in relation with the RGO/SPCE modified with the bioreceptor. This change in wettability suggests that once hybridized, the DNA molecules did not detach from the electrode surface, validating the XPS findings.

Surface accessibility is a crucial aspect impacted by wettability. The ability of the electrode surface to be wetted by biological samples, including DNA-containing solutions, is directly influencing the final performance of the detection device. An optimally wettable surface ensures uniform and efficient coverage, thereby enhancing the likelihood of successful RUNX2/ALP biomolecule-binding events, ensuring a stable and uniform attachment for reliable and reproducible sensor performance.

Tailoring wettability properties provides control over the hydrophilicity or hydrophobicity of the electrode surface, influencing the interactions between the electrode and DNA molecules. This control is crucial for manipulating sensitivity and selectivity, serving as a critical element in final biosensor design. Wettability’s role extends to the reduction in non-specific binding, where appropriate wetting helps minimize unwanted molecules’ adhesion to the electrode surface.

Finally, in electrochemical signal generation, optimal wettability ensures efficient electron transfer at the electrode interface during electrochemical reactions. This seamless transfer of electrons is essential for transducing DNA-binding events into measurable electrical signals, ultimately enhancing the sensitivity and responsiveness of the detection platform.

### 3.4. Electrochemical Characterization

In the context of ALP and RUNX2 detection, the formation of dsDNA from ssDNA sequences is typically a result of the hybridization-based detection strategies commonly employed in assays. Initially, the probe sequences are meticulously designed to be complementary to specific target sequences associated with ALP and RUNX2. Without incorporating signal amplification strategies, the formed complementary strands that are introduced during the detection process hybridize with the initially adsorbed probes on the electrode surface. The formation of double-stranded biomolecule serves as a measurable signal indicative of the presence or activity of ALP or RUNX2. This detection strategy, reliant on nucleic acid hybridization, remains a widely employed approach in various nucleic acid-based assays for its features.

**Figure 7:**
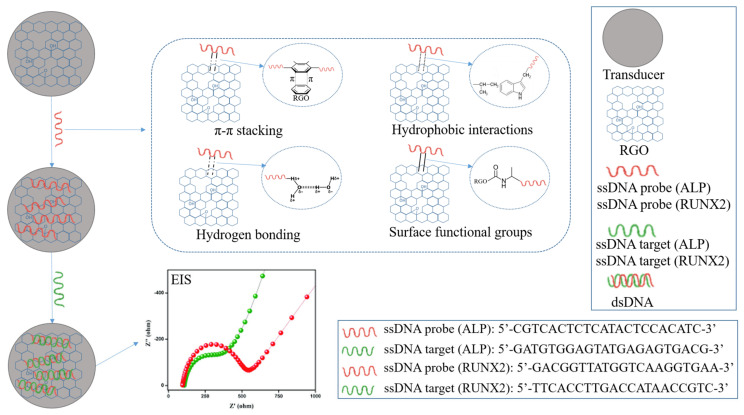
Schematic representation of ssDNA probe binding mechanism to RGO surface.

The binding mechanism between RGO and the ALP/RUNX2 probe bioreceptor involves a combination of non-covalent interactions, such as π–π stacking and hydrophobic interactions. Graphene and its derivatives, including RGO, exhibit a planar, sp^2^-hybridized carbon structure that facilitates strong π–π stacking interactions with the aromatic rings present in nucleotide bases, specifically adenine (A), thymine (T), guanine (G), and cytosine (C). These π–π stacking interactions occur due to the attractive forces between the π electron clouds of the nucleotide bases aromatic rings and the carbon atoms in the graphene structure, resulting in a stable and reversible binding. Additionally, the hydrophobic nature of RGO contributes to favorable hydrophobic interactions with the hydrophobic regions of the probe, consisting of nonpolar bases and the sugar-phosphate backbone. The presence of oxygen-containing functional groups on RGO, including hydroxyl and epoxy groups, further facilitates hydrogen bonding with functional groups on the ALP/RUNX2 bioreceptor, enhancing binding affinity. Furthermore, the negatively charged phosphate groups in the probe molecule may interact with positively charged regions on the RGO surface through electrostatic forces. This intricate combination of π–π stacking, hydrophobic interactions, surface functional groups, and charge interactions contributes to the binding mechanism, crucial for the successful immobilization of the ALP/RUNX2 probe bioreceptor on the RGO surface (Figure 7). This interaction forms a stable platform essential for biosensing applications, contributing to the biosensor’s selectivity and sensitivity in detecting target biomolecules.

#### 3.4.1. Detection of ALP Biomarker

**Figure 8:**
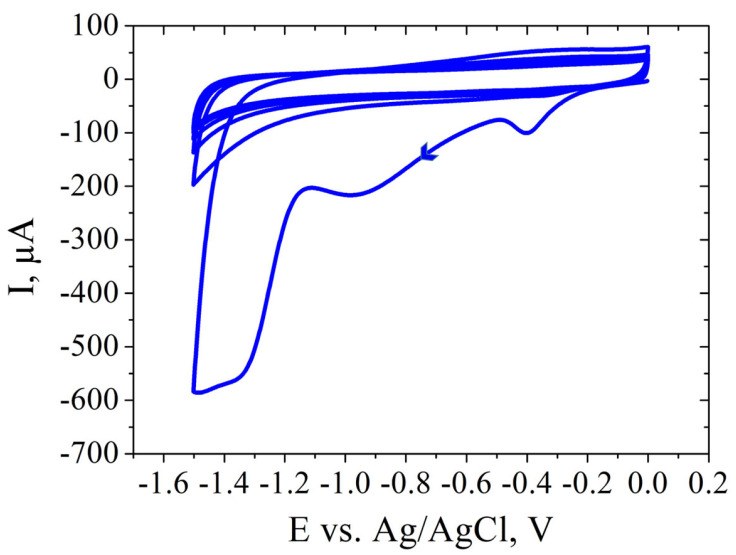
CV showing the electrochemical reduction in 0.5 M KCl of GO/SPCE.

SPCE modification was performed after electrochemical cleaning of the electrode. Applying PBS before GO modification serves to enhance electrochemical behavior and ensure biosensor reproducibility. The PBS treatment optimizes SPCEs by improving the peak-to-peak separation and reducing the charge transfer resistance (Rct). It also increases the hydrophilicity of the carbon surface, aiding GO adhesion. The addition of a small PBS volume on the working electrode modifies the wetting properties, preventing the GO solution from spreading beyond the working electrode borders. This step proves crucial for reproducible GO coating, ensuring consistent biosensor performance [39]. GO dispersion was used to coat the working electrode, then it was reduced electrochemically by performing CV in the presence of KCl solution (Figure 8).

**Figure 9:**
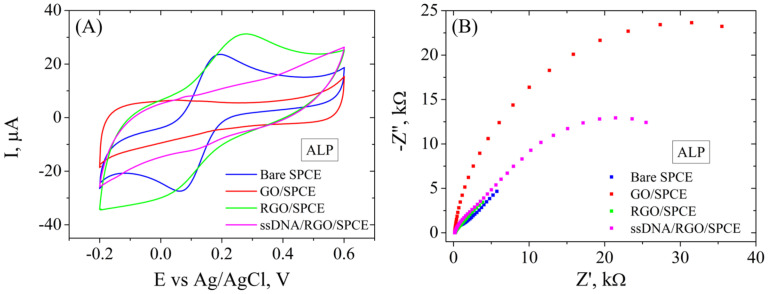
CV (**A**) and EIS Nyquist plot (**B**) recorded in 1 mM [Fe(CN)_6_]^3−/4−^, 0.1 M KCl, for bare SPCE, GO/SPCE, RGO/SPCE, and ALP ssDNA/RGO/SPCE.

The immobilization of the ALP bioreceptor was achieved by incubating the modified electrode in ssDNA probe solution overnight. Each step of these modifications was recorded electrochemically by CV and EIS (Figure 9) in the presence of 1 mM [Fe(CN)_6_]^3−/4−^ redox system. CV measurements (Figure 9A) show the typical properties for the bare electrode after electrochemical cleaning, i.e., well-defined redox peaks with a low peak-to-peak potential separation and a moderate current intensity. The CV curve changes drastically after GO deposition on the SPCE, showing no oxidoreduction peaks and a very low current intensity due to the poor conductive properties of this material. However, the electrochemical reduction of GO involved the elimination of functional groups from the GO surface, as was indicated by the aforementioned Raman spectroscopy and contact angle measurements, leading to a higher current intensity recorded for this material, CV showing again the characteristic redox peaks and superior electrochemical properties. The immobilization of the ALP probe blocked the electrode surface, causing a significant decrease in peak intensity. These results are correlated with EIS measurements (Figure 9B) that show a very low charge transfer resistance (Rct) for the bare electrode and after electrochemical reduction of GO, as can be seen by the absence of the semicircle in the Nyquist plot, while the first modification with GO shows a high Rct. After functionalization with the ALP bioreceptor, Rct increased significantly compared to RGO/SPCE, confirming the blocking of the electron transfer between the electrolyte solution and the electrode surface.

**Figure 10:**
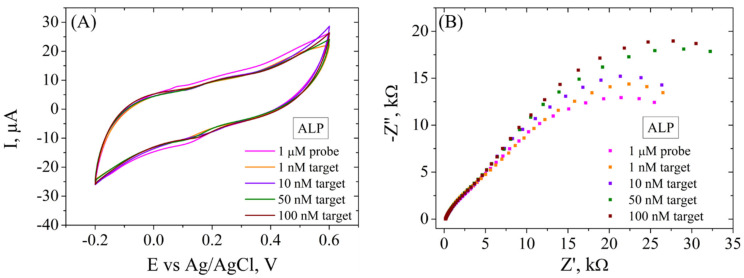
CV (**A**) and EIS Nyquist plot (**B**) recorded in 1 mM [Fe(CN)_6_]^3−/4−^, 0.1 M KCl, for ssDNA/RGO/SPCE and after hybridization with 1, 10, 50, and 100 nM ALP target.

The electrochemical platform was tested for the determination of various concentrations of ALP biomarker by electrochemical methods (Figure 10). In CV (Figure 10A), it is observed that the incubation of the functionalized electrode with 1, 10, 50 and 100 nM DNA target caused a decrease in the current intensity (from 5 to 2 µA), but not a significant difference is observed for all concentrations. However, EIS (Figure 10B), being a more sensitive electrochemical tool, records variations in Rct after incubation in each ALP target concentration, recording an increase after each step (from 34.1 kΩ to 51.2 kΩ), indicating that the hybridization between probe and each target molecule was successful and the dsDNA formed did not detach from the electrode surface, in agreement with XPS indications and contact angle measurements. Moreover, there are two semicircles observed after incubation of the electrode with both DNA probe and DNA target, and the first one does not change in size following incubation with any target concentration, suggesting that the system has a component with properties that are not suffering modifications.

#### 3.4.2. Detection of RUNX2 Biomarker

**Figure 11:**
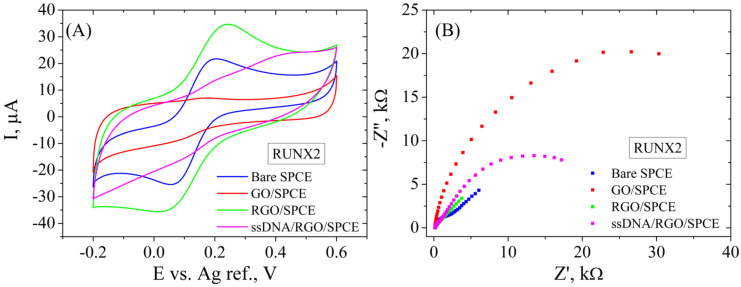
CV (**A**) and EIS Nyquist plot (**B**) recorded in 1 mM [Fe(CN)_6_]^3−/4−^, 0.1 M KCl, for bare SPCE, GO/SPCE, RGO/SPCE, and RUNX2 ssDNA/RGO/SPCE.

In the second case, SPCE modification was performed by the same protocol mentioned above, and the electrochemical properties are similar for the RGO/SPCE functionalized with the RUNX2 bioreceptor (Figure 11). In short, CV (Figure 11A) shows a low current intensity for GO/SPCE that increases substantially after the reduction process. The incubation with the RUNX2 probe leads to lower redox currents, suggesting in this case too that the DNA molecule is blocking the electrode surface. Again, EIS measurements (Figure 11B) support these results, showing again a very low Rct for the unmodified SPCE that increases significantly after GO deposition, shown as a large semicircle in the Nyquist plot. The GO electrochemical reduction causes the disappearance of the semicircle due to the low Rct, while the functionalization with the bioreceptor leads to a high resistance to the electron transfer between the redox probe and the surface of the electrode.

**Figure 12:**
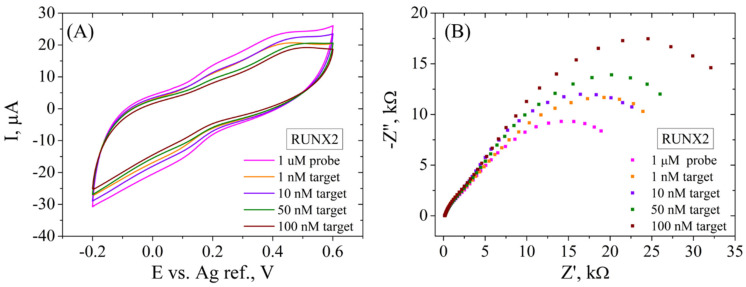
CV (**A**) and EIS Nyquist plot (**B**) recorded in 1 mM [Fe(CN)_6_]^3−/4−^, 0.1 M KCl, for ssDNA/RGO/SPCE and after hybridization with 1, 10, 50, and 100 nM RUNX2 target.

When this platform was tested for the electrochemical detection of the RUNX2 biomarker (Figure 12), better results were obtained compared to the ALP detection. In this case, CV results (Figure 12A) show a successive decrease of the current intensity (from 8 to 2 µA) after incubation of the functionalized SPCE with 1, 10, 50 and 100 nM DNA target, indicating that dsDNA was obtained from the hybridization between the RUNX2 probe and the target molecule. The EIS recordings (Figure 12B) present a higher sensitivity in the electrochemical signal compared to ALP detection, but a similar trend nonetheless. Specifically, a significant increase in Rct is observed after hybridization with each RUNX2 target concentration (from 25.3 kΩ to 48.1 kΩ), as indicated by the increase in size of the second semicircle in the Nyquist plot. As in the ALP detection case, the first semicircle does not change in size, indicating a constant phase in the system.

## 4. Conclusions

In summary, we have proposed a simple and efficient technique consisting of modifying commercial electrodes with electrochemically reduced graphene oxide as the starting point of developing a promising detection platform for osteogenic biomarkers, such as ALP and RUNX2, outlining the essential parameters necessary for this design and offering a superior alternative for the current laborious, expensive, and time-consuming approaches. The modification and functionalization of the electrodes were visualized with SEM, Raman spectroscopy, XPS, and contact angle measurements. In addition, CV and EIS were the electrochemical tools employed to characterize the system and to detect the specific biomarkers. All the characterization techniques confirmed successful SPCE modification with GO and its electrochemical reduction into RGO. SEM showed changes in the electrode surface morphology, while Raman spectroscopy confirmed that the structural properties were modified after the electrochemical procedure applied to GO/SPCEs, indicating the removal of oxygenated functional groups from the modified electrode surface. XPS results provided insightful details regarding the surface composition and chemical states of SPCE modifications, confirming the successful immobilization of the probe onto the RGO surface and the integration of target biomarkers. Contact angle investigations not only endorsed these findings but also showed changes in the wettability properties after immobilization of the bioreceptor and the hybridization with the complementary target, respectively, supporting the electrochemical characterization results. Both CV and EIS show that RGO-modified electrodes have great potential in detecting osteogenic biomarkers up to 1 nM. Moreover, our study shows that the RGO platform has a higher sensitivity towards the detection of RUNX2 compared to the ALP biomarker. Therefore, further investigations are necessary and ongoing in order to improve in our future work the sensitivity of the initial graphene-based platform for both osteogenic biomarkers presented and to provide an in-depth analysis of the final biosensor parameters so as to increase the potential of this system to be fabricated at industrial scale.

## Data Availability

The raw data supporting the conclusions of this article will be made available by the authors on request.
